# The potential risk of ventilator-induced lung injury from five different PEEP titration techniques in ARDS

**DOI:** 10.3389/fmed.2025.1642064

**Published:** 2025-08-29

**Authors:** Yuda Sutherasan, Chayanon Songsomboon, Kridsanai Gulapa, Detajin Junhasavasdikul, Pongdhep Theerawit

**Affiliations:** ^1^Division of Pulmonary and Pulmonary Critical Care Medicine, Department of Medicine, Faculty of Medicine Ramathibodi Hospital, Mahidol University, Bangkok, Thailand; ^2^Division of Critical Care Medicine, Department of Medicine, Faculty of Medicine, Ramathibodi Hospital, Mahidol University, Bangkok, Thailand

**Keywords:** acute respiratory distress syndrome, ventilator-induced lung injury, transpulmonary pressure, electrical impedance tomography, PEEP titration, esophageal pressure, lung compliance

## Abstract

**Introduction:**

The optimal positive end-expiratory pressure (PEEP) in acute respiratory distress syndrome (ARDS) remains uncertain. This study compared the PEEP levels using five distinct titration methods to assess potential ventilator-induced lung injury (VILI).

**Methods:**

This study included 21 patients with moderate to severe ARDS who were monitored using esophageal balloon manometry and electrical impedance tomography (EIT). A recruitment maneuver followed by decremental PEEP titration was performed. Optimal PEEP (OP) was assessed using five criteria: highest respiratory system compliance (C_RS_), highest lung compliance (C_L_), end-expiratory transpulmonary pressure (P_tp_ee_direct_) ≥ 0 cm H_2_O, elastance-derived end-inspiratory transpulmonary pressure (P_tp_ei_derived_) ≤ 25 cm H_2_O, and EIT-based analysis balancing the degree of overdistention and lung collapse.

**Results:**

Significant differences in OP were observed across the methods (*p* = 0.001): C_RS_ 8.0 cmH₂O (8.0,13.9); C_L_ 9.8 cmH₂O (8.0,14.0); P_tp_ee_direct_ ≥ 0 cmH₂O 14.0 cm H₂O (11.9,17.9); P_tp_ei_derived_ ≤ 25 cmH₂O 12.0 cmH₂O (10.0,13.9); EIT balancing the degree of overdistention and lung collapse 13.01 cmH₂O (9.88,14.78). The OP guided by P_tp_ee_direct_ of ≥ 0 cm H_2_O is significantly higher than OP by the highest C_RS_ (*p* = 0.001) and the highest C_L_ (*p* = 0.002), and met the overdistension criteria, namely plateau pressure > 30 cm H_2_O and the highest percentage of overdistension by EIT. The PEEP guided by C_RS_ had a higher potential risk of lung collapse, reflected by the negative value of P_tp_ee_direct_ and a higher percentage of lung collapse by EIT.

**Conclusion:**

Transpulmonary pressure-guided PEEP titration yielded higher PEEP levels, while C_RS_-guided PEEP was lower and associated with a higher risk of collapse. Overdistension and collapse varied with the chosen PEEP method. In patients with moderate to severe ARDS, OP can vary depending on the method of assessment.

## 1 Introduction

Over the past several decades, the decline in ARDS mortality has been largely attributed to lung-protective mechanical ventilation strategies designed to minimize ventilator-induced lung injury (VILI). VILI primarily results from excessive lung stress and strain, manifesting as volutrauma, barotrauma, atelectrauma, and biotrauma (1, 2).

Optimizing positive end-expiratory pressure (PEEP) in ARDS mitigates atelectrauma and prevents VILI (3, 4). Understanding each patient’s unique physiology and adjusting mechanical ventilation settings using advanced monitoring tools may enhance outcomes. In 1975, Suter et al. performed the first study on optimal PEEP (OP), defining it as the level that maximized oxygen transport and respiratory system compliance (C_RS_) while minimizing dead space, based on arterial oxygenation, hemodynamics, and respiratory mechanics measurements (3).

Randomized controlled trials (RCTs) have explored various methods for identifying OP, including the ARDS Network PEEP/Fraction of inspired oxygen (FiO₂) tables, compliance-based titration, and recruitment-maneuver (RM)-guided adjustments. However, the absence of consensus and the wide variation in PEEP practices across institutions complicate the interpretation of the overall efficacy of these strategies in ARDS (4).

Recent studies have introduced personalized PEEP-titration strategies for ARDS (5–10). targeting physiological variables such as driving and plateau pressures, transpulmonary-pressure (P_tp_) monitoring, and bedside electrical impedance tomography (EIT) (5–10).

Hickling et al. demonstrated that decremental PEEP titration—guided by optimal C_RS_—was more effective at opening ARDS lungs than incremental titration (6). This technique is mainly used following the RM. Esophageal balloon catheters guide OP settings by accounting for lung stress and strain through P_tp_ monitoring (8). Maintaining a direct end-expiratory transpulmonary pressure (P_tp_ee_direct_) near 0 cm H₂O was associated with improved survival compared with targeting more positive or negative pressures (7). However, pooled mortality did not differ significantly between mechanics-based PEEP strategies and ARDS Network PEEP/FiO₂ tables in a meta-analysis (4).

EIT has recently been proposed for PEEP titration in ARDS (9, 11). This technique has shown benefits in reducing Sequential Organ Failure Assessment (SOFA) scores compared with guidance provided by the ARDS Network PEEP/FiO₂ table (11, 12). Furthermore, EIT can quantify regional overdistension and collapse (10, 13). However, the extent of lung overdistension and collapse at a given low tidal volume and optimal PEEP—when guided by different techniques—remains unclear.

We hypothesized that varying PEEP targets would result in differing degrees of lung overdistension and atelectasis. To test this, we enrolled mechanically ventilated ARDS patients.

Our primary objective was to compare OP values determined by five distinct methods:

1. Best C_RS_
2. Best lung compliance (C_L_)
3. Lowest PEEP yielding P_tp_ee_direct_ ≥ 0 cm H₂O
4. PEEP corresponding to an upper limit of elastance derived end-inspiratory transpulmonary pressure (P_tp_ei_derived_) ≤ 25 cm H₂O
5. EIT-based balance of regional overdistension and collapse.

Furthermore, we aimed to determine the degree of overdistension and collapse, including lung mechanic parameters. We compared them among five PEEP titration techniques guided by respiratory or lung mechanics, P_tp_, or EIT during decremental PEEP titration in ARDS patients.

## 2 Materials and methods

We conducted a prospective physiological study in ARDS patients admitted to the medical ICUs at Ramathibodi Hospital, Bangkok, between June 2020 and April 2021.

### 2.1 Patients

Patients aged 18 years or older who had moderate to severe ARDS according to the Berlin classification were included in the study (14). Exclusion criteria included contraindications to EIT (e.g., presence of a pacemaker or automatic intracardiac defibrillator), pregnancy, ongoing intercostal drainage, and chronic obstructive lung disease. The study was approved by the Institutional Review Board of the Faculty of Medicine, Ramathibodi Hospital (ID MURA2020/751), and written informed consent was obtained from patients’ next of kin. We also confirmed that the data were anonymized and maintained confidentially in compliance with the Declaration of Helsinki.

### 2.2 Measurements and experimental protocol

**Figure 1:**
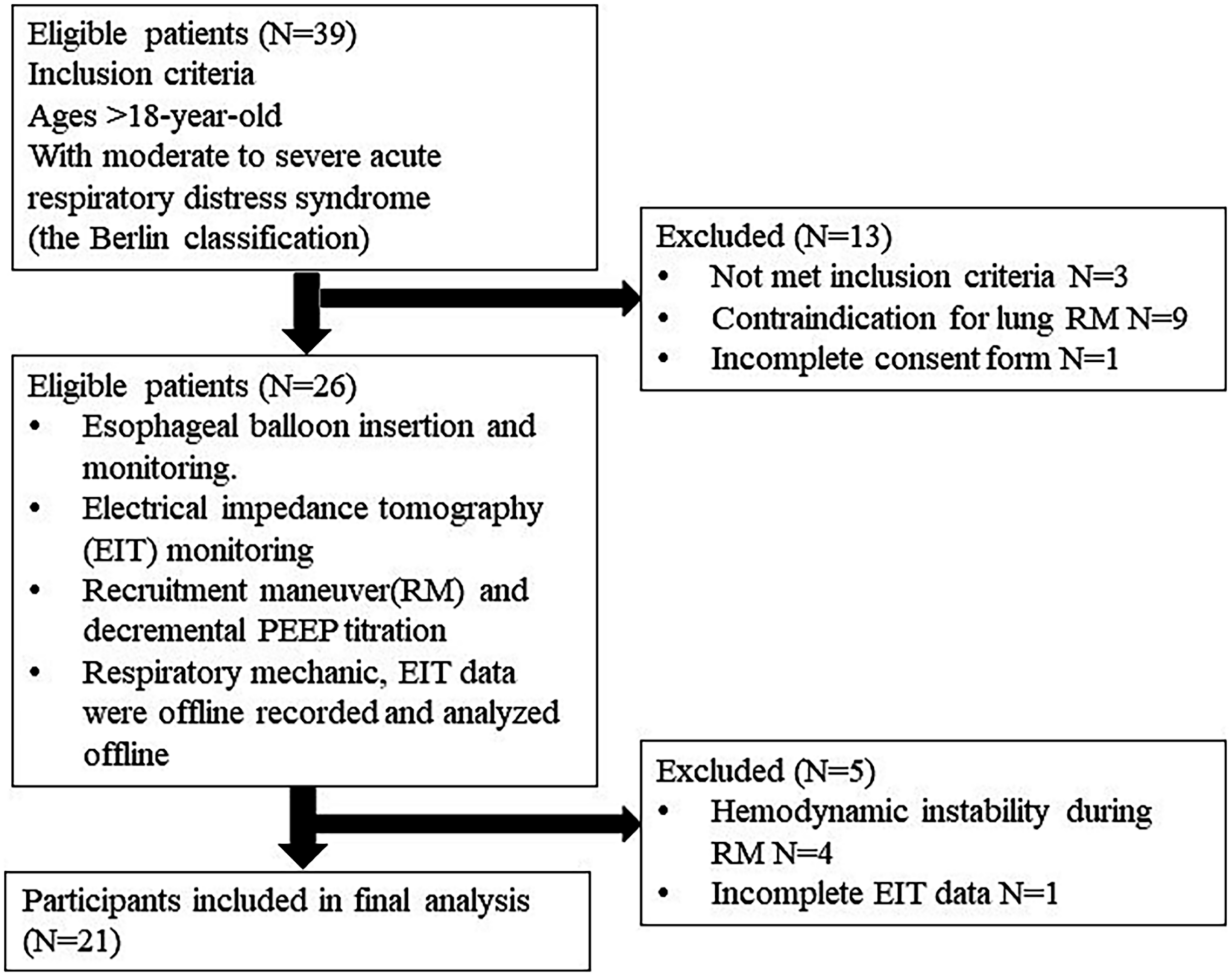
The protocol flow chart.

All patients were ventilated in the supine position on a Hamilton G5 ventilator (Hamilton Medical, Bonndorf, Switzerland) equipped with a dual-limb circuit and heated humidifier. They were deeply sedated with midazolam, propofol, or fentanyl and received a continuous cisatracurium infusion to suppress spontaneous breathing. Baseline characteristics, ARDS etiology, respiratory variables, ventilator settings, and PaO₂/FiO₂ ratios were recorded. The protocol flow chart is shown in Figure 1.

#### 2.2.1 Esophageal pressure monitoring

With the head of the bed elevated to 30 degrees, an esophageal balloon catheter (Cooper Surgical, Trumbull, CT) was advanced to a depth of 35–40 cm and positioned in the lower third of the esophagus. Patients were then returned to the supine position. The balloon was inflated with 1–2 mL of air, and correct placement was confirmed using the end-expiratory occlusion technique (15). The respiratory mechanics and esophageal pressure parameters were continuously recorded and exported from the ventilator for offline interpretation.

#### 2.2.2 Electrical impedance tomography

EIT measurements were obtained with a PulmoVista 500 device (Dräger Medical, Lübeck, Germany). The electrode belt was placed between the fifth and sixth intercostal spaces to detect regional ventilation. The resulting EIT plethysmogram—a waveform generated by summing all pixels within the region of interest (ROI) and plotting the relative impedance change over time—reflects the volume of air moving in and out of each ROI. Costa et al. developed a method for estimating the percentages of alveolar collapse and overdistension during decremental PEEP maneuvers by calculating pixel compliance (see Supplementary Figure 1) (10). The percentages of alveolar collapse and overdistension determined by EIT were recorded. Additionally, breath-by-breath EIT data were collected and analyzed offline.

#### 2.2.3 Recruitment maneuver and decremental PEEP titration

Hemodynamic stability was confirmed; Fluid status was assessed and, when indicated, optimized prior to the PEEP trial to minimize the risk of hemodynamic deterioration during the RM and subsequent decremental PEEP titration. Before each PEEP trial step, we performed a two-minute RM in pressure-controlled ventilation (PCV) with an inspiratory pressure of 25 cm H₂O above a PEEP of 20 cm H₂O, a respiratory rate of 10 breaths per minute, and an inspiratory-to-expiratory ratio of 1:1. After RM, PCV was maintained with a fixed inspiratory pressure of 15 cm H₂O above PEEP, and PEEP was decreased from 20 cm H₂O to 8 cm H₂O in 2 cm H₂O steps at one-minute intervals. At zero flow, alveolar and proximal airway pressures equilibrate, permitting plateau pressure and PEEP to serve as surrogates for inspiratory and expiratory alveolar pressures, respectively. We monitored the flow–time curve to ensure airflow returned to zero at the end of both inspiration and expiration, thereby using end-inspiratory airway pressure (P_aw_ei_) as plateau pressure and confirming the absence of intrinsic PEEP. After 1 min at each PEEP level, the following parameters were recorded:

1. Exhaled tidal volume (Tv_exh_) per predicted body weight (PBW)
2. P_aw_ei_ or plateau pressure
3. End-expiratory airway pressure (P_aw_ee_)
4. End-inspiratory esophageal pressure (P_eso_ei_)
5. End-expiratory esophageal pressure (P_eso_ee_)
6. Heart rate (HR)
7. Stroke volume (SV)
8. Cardiac output (CO)
9. Pulse pressure variation (PPV)

The following techniques were used for the assessment of P_tp_, lung elastance (E_L_), and chest wall elastance (E_CW_):

1. P_tp_ee_direct_ = P_aw_ee_ – P_eso_ee_ (8)
2. Direct measurement of end-inspiratory transpulmonary pressure (P_tp_ei_direct_) = P_aw_ei_ -P_eso_ei_ (8)
3. E_L_ = (P_tp_ei___direct_ – P_tp_ee_direct_)/ Tv_exh_ (16)
4. E_CW_ = (P_eso_ei_ - P_eso_ee_)/Tv_exh_
5. Respiratory system elastance (E_RS_) = (P_aw_ei_ -P_aw_ee_)/ Tv_exh_
6. P_tp_ei_derived_ = P_aw_ei_ x E_L_/E_RS_ (17).

## 3 Outcomes

The main objective was to compare the OP chosen using five different methods and assess the extent of overdistention and collapse associated with each method. The OP was determined from the following five methods:

1. The “best” Crs
2. The “best” C_L_
3. The lowest PEEP providing the P_tp_ee_direct_ ≥ 0 cm H_2_O
4. The PEEP providing the upper limit of P_tp_ei_derived_ ≤ 25 cm H_2_O
5. The PEEP from the EIT analysis balances the degree of overdistention and lung collapse (10) (Supplementary Figure 1).

The following criteria characterized the potential risk of VILI at the optimum PEEP:

1. The airway plateau pressure > 30 cm H_2_O
2. The lung stress by P_tp_ei_derived_ > 25 cm H_2_O
3. The percentage of overdistension by EIT
4. The negative P_tp_ee_direct_
5. The percentage of lung collapse by EIT.

## 4 Statistical analysis

Regarding the OP levels, we identified them from five distinct techniques. Sample size was calculated *a priori* in G*Power for a one-way ANOVA with fixed effects (omnibus test). Assuming a large effect size (*f* = 1.0), *α* = 0.05, 80% power, and five independent groups, a total of 20 participants was required. Under these parameters, the noncentrality parameter (*λ*) was 20.00, the critical *F*-value was 3.06 (df₁ = 4; df₂ = 15), and the achieved power was 0.88.

Data are presented as mean ± standard deviation (SD) or median (interquartile range [IQR]). The Student’s t-test was used to compare two continuous variables, and one-way ANOVA (F statistic) was applied for comparisons involving more than two groups. Repeated-measures ANOVA with Greenhouse–Geisser correction analyzed variables during PEEP titration. Nonparametric data were assessed using the Kruskal–Wallis test. A *p*-value < 0.05 was considered statistically significant. All analyses were performed with SPSS version 22.0 (IBM, Armonk, NY, USA).

## 5 Results

### 5.1 Baseline characteristics

Among thirty-nine patients, twenty-six were eligible. Four patients were excluded due to hemodynamic instability during RM, and one patient had incomplete EIT data. Twenty-one patients were included in the final analysis. The baseline characteristics of patients are shown in Table 1. According to the Berlin definition, all patients presented with moderate to severe ARDS. The mean PaO_2_/FiO_2_ ratio was 115.16 ± 28.17 mm Hg. The primary etiology of ARDS was severe pneumonia in most patients (90.9%). The mean tidal volume and respiratory compliances were 6.20 ± 1.05 mL/kg PBW and 30.5 ± 7.5 mL/cm H_2_O, respectively. The median Ecw/Ers was 0.18 (0.11, 0.27). The E_L_/E_RS_ and E_CW_/E_RS_ were constant during decremental PEEP titration, with *p*-values of 0.99 and 0.99, respectively, by the Kruskal-Wallis test.

**Table 1:** The baseline characteristics.

Patients’ baseline characteristics	All (*N* = 21)
Female, *n* (%)	11 (52.38)
Age, years	61 ± 19.96
BMI, kg/m^2^	23.62 + 3.67
Comorbidities, *n* (%)	
Immunocompromised	16 (72.72)
Hematologic malignancy	3(12.63)
Solid tumor	5(22.72)
Hypertension	8 (36.36)
Diabetis mellitis	5 (22.72)
Chronic kidney disease	7 (31.81)
APACHE II at 1st 24 h admission	26 ± 5.71
Type of ARDS, *n* (%)	
Intrapulmonary cause	20 (90.9)
Berlin definition ARDS severity, *n* (%)	
Moderate	17 (81%)
Severe	4(19%)
PaO_2_/FiO_2_ ratio	115.16 ± 28.17
PaCO_2_	37.49 ± 10.44
Baseline hemodynamic data	
SBP, mmHg	121.23 ± 20.64
DBP, mmHg	61.41 ± 9.03
MAP, mmHg	81.41 ± 10.57
Vasopressor during inclusion, *n* (%)	17 (81%)
Dose of norepinephrine, mcg/kg/min	0.12 ± 0.10
Arterial lactate, mmol/L	1.68 ± 1.26
PPV, %	8.67 ± 7.6
SVV, %	9.86 ± 8.62
CO, liters/min	6.44 ± 4.63

BMI, body mass index; APACHE, Acute Physiology And Chronic Health Evaluation; ARDS, Acute respiratory distress syndrome; PaO_2_, Partial pressure of oxygen; FiO_2_, fraction of inspired oxygen; PaCO_2_, Partial Pressure of Carbon Dioxide; SBP, systolic blood pressure; DBP, diastolic blood pressure; PPV, pulse pressure variation; SVV, stroke volume variation; CO, cardiac output.

The mean SpO₂/FiO₂ ratio increased from 146.97 ± 58.20 pre-recruitment to 150.29 ± 58.79 immediately post-RM, although this early change was not statistically significant after adjustment for multiple comparisons. At all subsequent PEEP levels (20 to 8 cm H₂O), the SpO₂/FiO₂ ratio remained significantly higher than pre-RM (Bonferroni-adjusted *p* < 0.05 for each comparison) (Supplementary Table 1).

**Figure 2:**
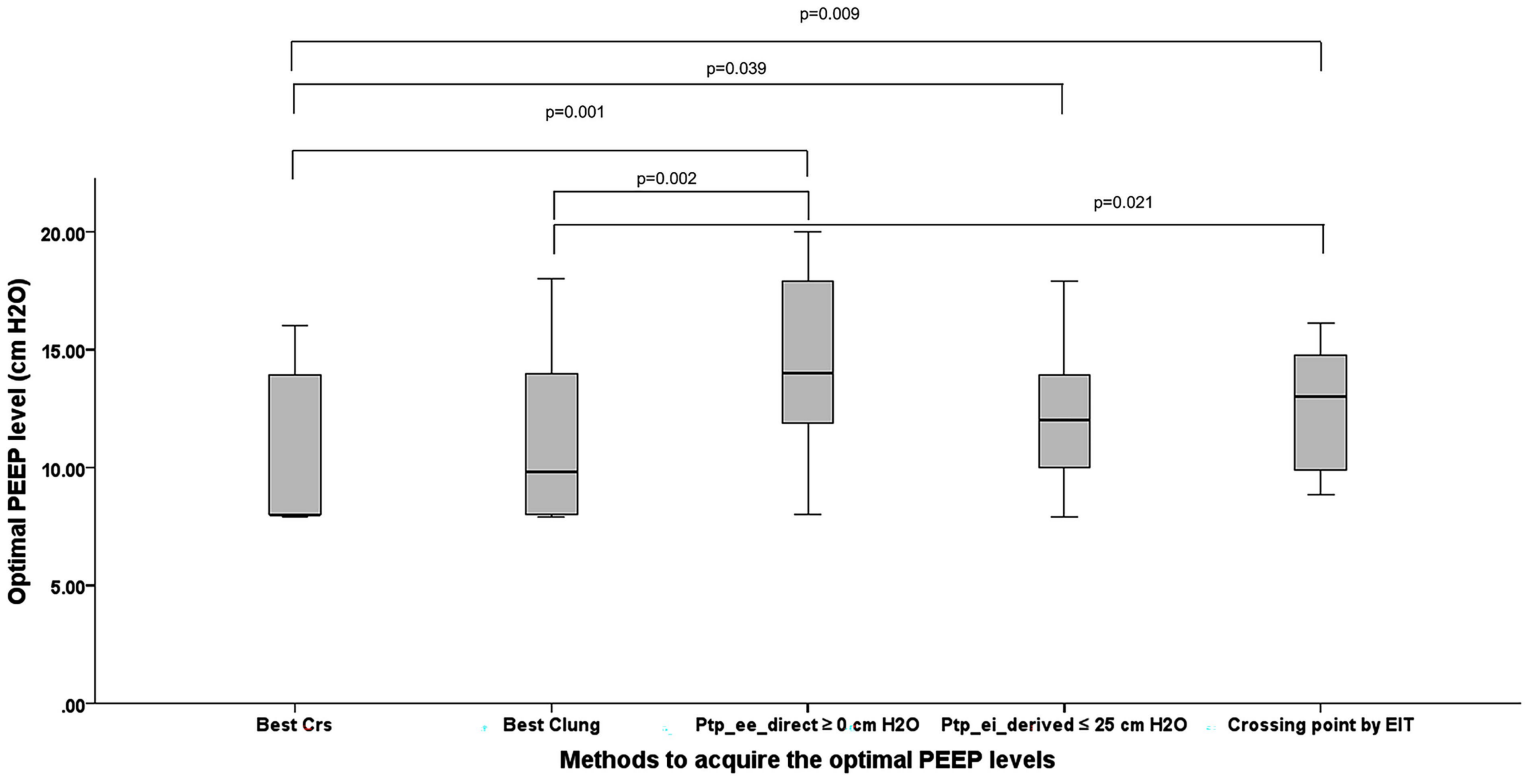
The comparison among PEEP acquired from each technique demonstrates statistical differences between the methods (*p* = 0.001). PEEP, positive end-expiratory pressure; Best Crs, the optimum PEEP (OP) acquired from the highest respiratory system compliance; Best Clung, the OP acquired from the highest lung compliance; P_tp_ee_direct_ ≥0 cm H_2_O, The lowest PEEP providing the direct measurement of end-inspiratory transpulmonary pressure ≥0 cm H_2_O; P_tp_ei_derived_ ≤25 cm H_2_O, The PEEP providing the upper limit of derived end inspiratory transpulmonary pressure ≤25 cm H_2_O; Crossing point by EIT, The PEEP from the electrical impedance tomography analysis balancing the degree of overdistention and lung collapse.

We found a statistically different OP level among the five targets (*p* = 0.001) (Figure 2). When titrated to maximize C_RS_, the median PEEP was 8.00 cmH₂O (8.00, 13.90), whereas titration for optimal C_L_ yielded a slightly higher median of 9.80 cmH₂O (8.00, 14.00). Targeting P_tp_ee_direct_ of ≥ 0 cmH₂O resulted in the highest median PEEP of 14.00 cmH₂O (11.90, 17.90), while limiting the P_tp_ei_derived_ to ≤ 25 cmH₂O produced a median of 12.00 cmH₂O (10.00, 13.90). Finally, the EIT crossing-point method selected an intermediate median PEEP of 13.01 cmH₂O (9.88, 14.78). The OP guided by P_tp_ee_direct_ of ≥ 0 cm H_2_O is significantly higher than OP by the highest respiratory system compliance (*p* = 0.001) and the highest lung compliance (*p* = 0.002).

### 5.2 The potential risk of overdistension

**Figure 3:**
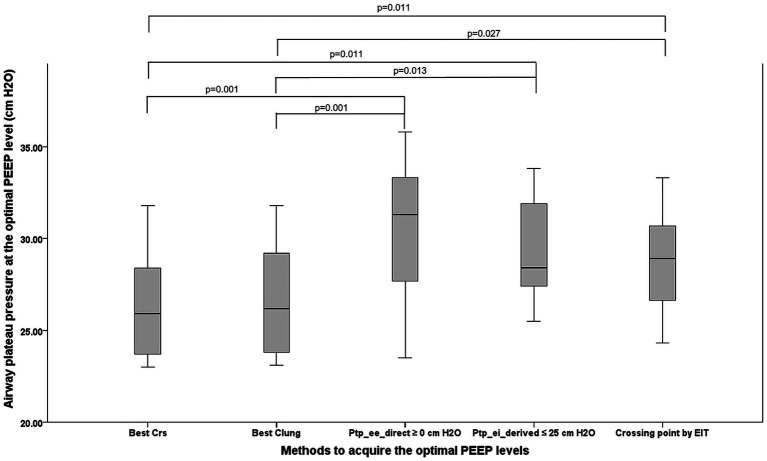
The picture demonstrates the airway plateau pressure occurring from the OP acquired from five methods. PEEP, positive end-expiratory pressure; OP, optimum positive end expiratory pressure, Best Crs, the OP acquired from the highest respiratory system compliance; Best Clung, the OP acquired from the highest lung compliance; P_tp_ee_direct_ ≥0 cm H_2_O, The lowest PEEP providing the direct measurement of end-inspiratory transpulmonary pressure ≥0 cm H_2_O; P_tp_ei_derived_ ≤25 cm H_2_O, The PEEP providing the upper limit of derived end inspiratory transpulmonary pressure ≤25 cm H_2_O; Crossing point by EIT, The PEEP from the electrical impedance tomography analysis balancing the degree of overdistention and lung collapse.

The OP guided by P_tp_ee_direct_ ≥ 0 cm H₂O met overdistension criteria, with a median plateau pressure of 31.30 cm H₂O (27.70, 33.30) (Figure 3). In comparison, the median plateau pressures for OP guided by C_RS_, C_L_, P_tp_ei_derived_, and EIT were 25.90 cm H₂O (23.70, 28.40), 26.20 cm H₂O (23.80, 29.20), 28.40 cm H₂O (27.40, 31.90), and 28.93 cm H₂O (26.65, 30.68), respectively. Significant differences were found between techniques: C_RS_ vs. P_tp_ee_direct_ (*p* = 0.001), C_RS_ vs. P_tp_ei_derived_ (*p* = 0.011), C_RS_ vs. EIT (p = 0.011), C_L_ vs. P_tp_ee_direct_ (*p* = 0.001), C_L_ vs. P_tp_ei_derived_ (*p* = 0.013), and C_L_ vs. EIT (*p* = 0.027).

Lung stress in ARDS refers to the P_tp_- the pressure difference between the alveolar space and the pleural space that actually distends the lung tissue. The lung stress, which was calculated from the elastance-derived calculation (P_tp_ei_derived_ = P_aw_ei_ x E_L_/E_RS_), was compared across the PEEP titration methods. The highest mean P_tp_ei_derived_ occurred with P_tp_ee_direct_ guidance (24.74 ± 4.79 cm H₂O); meanwhile, the lowest mean value appeared in PEEP directed by the best C_L_ (21.00 ± 4.54 cm H_2_O), which was significantly lower (*p* = 0.042). C_RS_ guided lung stress (21.04 ± 4.17 cm H₂O) was also significantly lower than that under P_tp_ee_direct_ guidance (*p* = 0.046).

For EIT-derived overdistension, the P_tp_ee_direct_ guided method yielded the highest median percentage (17%; 6, 22), significantly exceeding the best C_RS_ [0% (0, 8.5); *p* = 0.002] and best C_L_ [5% (0, 9); *p* = 0.004] methods. P_tp_ei_derived_- and EIT-guided techniques produced 10.5% (0.0, 22.0) and 6.8% (4.0, 10.8), respectively, with no further significant differences.

EIT overdistension percentage correlated strongly with peak airway pressure in PCV mode (*r* = 0.67; *p* < 0.001), and with elastance-derived lung stress (*r* = 0.48; *p* < 0.001), but not with driving pressure.

### 5.3 The potential risk of lung collapse

It has been suggested that adjusting PEEP based on P_tp_ee_direct_ can recruit atelectatic lung units in dependent regions (18). Accordingly, we used P_tp_ee_direct_ to assess the potential risk of lung collapse, and employed EIT analysis to quantify the percentage of collapse (10).

**Figure 4:**
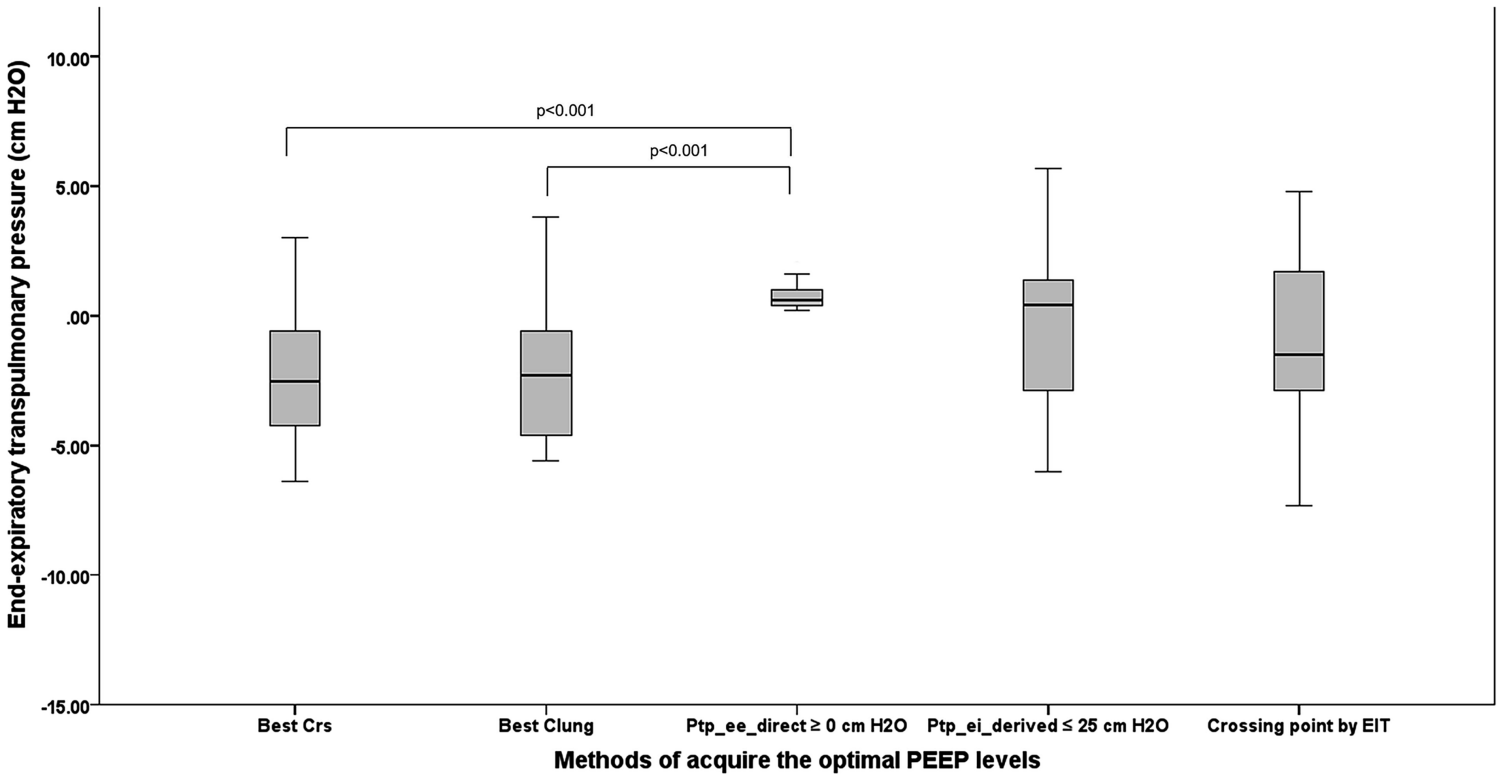
The comparison among five techniques to acquire the OP regarding lung collapse in terms of the direct measurement of P_tp_ee_direct_. PEEP, positive end-expiratory pressure; OP, optimum positive end expiratory pressure; P_tp_ee_direct_, end-inspiratory transpulmonary pressure; Best Crs, the OP acquired from the highest respiratory system compliance; Best Clung, the OP acquired from the highest lung compliance; P_tp_ee_direct_ ≥0 cm H_2_O, The lowest PEEP providing the direct measurement of end-inspiratory transpulmonary pressure ≥0 cm H_2_O; P_tp_ei_derived_ ≤25 cm H_2_O, The PEEP providing the upper limit of derived end inspiratory transpulmonary pressure ≤25 cm H_2_O; Crossing point by EIT, The PEEP from the electrical impedance tomography analysis balancing the degree of overdistention and lung collapse.

Figure 4 compares P_tp_ee_direct_ across the five PEEP-titration methods. Negative median P_tp_ee_direct_ values—indicative of potential lung collapse—were observed with PEEP guided by C_RS_ [−2.3 cm H₂O (−4.2,–0.8)], C_L_ [−2.3 cm H₂O (−4.5,–0.8)], and EIT [−1.5 cm H₂O (−2.9, 1.7)]. These methods yielded significantly lower P_tp_ee_direct_ than PEEP set by direct P_tp_ee_direct_ itself (*p* < 0.001 vs. C_RS_; *p* < 0.001 vs. C_L_).

The greatest risk of lung collapse occurred with C_RS_-guided PEEP, which had a median P_tp_ee_direct_ of −2.3 (−4.2, −0.8) cm H_2_O and a median EIT-measured collapse of 13% (6, 19). In contrast, P_tp_ee_direct_-guided PEEP showed the lowest collapse risk, with a median P_tp_ee_direct_ of 0.4 cm H₂O (0.6, 1.0) and an EIT collapse of 1.5% (0.0, 7.5).

**Figure 5:**
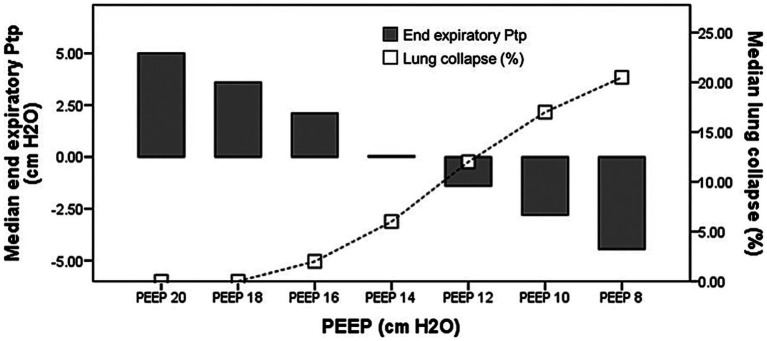
The relationship between P_tp_ee_direct_ and the percentage of collapse regarding. PEEP titration. P_tp_ee_direct_, the direct measurement of end-inspiratory transpulmonary pressure; PEEP, positive end expiratory pressure.

P_tp_ee_direct_ correlated negatively with EIT-derived collapse percentage (*r* = −0.62, *p* < 0.001). Figure 5 illustrates the relationship between P_tp_ee_direct_ and the percentage of collapse by EIT during PEEP titration; the P_tp_ee_direct_ associated with zero lung collapse on EIT was 5.15 cm H₂O (1.90, 6.70).

### 5.4 Directly measured vs. elastance-derived transpulmonary pressure

Both P_tp_ei_direct_ and P_tp_ei_derived_ decreased during decremental PEEP titration. Repeated-measures ANOVA showed that P_tp_ei_direct_ was significantly lower than P_tp_ei_derived_ (*p* < 0.001; Supplementary Figure 2), and this difference remained consistent throughout titration (*p* = 0.954), with a mean difference of 10.91 ± 3.82 cm H₂O.

### 5.5 Impact on hemodynamics

No significant changes in HR, SV, or CO were observed during decremental PEEP titration (*p* = 1.000, 0.992, and 0.990, respectively). PPV declined during titration but did not reach statistical significance (*p* = 0.375). Kruskal–Wallis analysis showed no significant differences in HR, CO, or PPV at OP across the five methods (*p* = 0.987, 0.992, and 0.975, respectively) (Supplementary Table 1).

## 6 Discussion

We conducted a prospective observational study in a homogeneous cohort of mechanically ventilated ARDS patients. Strengths of the study included: (1) direct comparison of multiple PEEP-titration methods; (2) evaluation of potential associations with overdistension and collapse; and (3) incorporation of diverse monitoring modalities, including esophageal pressure and EIT.

The main findings are: (1) OP differed across the five titration methods; (2) P_tp_ee_direct_ -guided PEEP (≥0 cm H₂O) was higher than PEEP determined by C_RS_ or C_L_; (3) P_tp_ee_direct_ -guided PEEP exceeded overdistension thresholds (plateau pressure >30 cm H₂O) and produced the highest percentage of overdistension; and (4) C_RS_ showed a greater potential risk of lung collapse, evidenced by negative end-expiratory P_tp_ values and a higher EIT-derived collapse percentage.

PEEP titration in ARDS is essential for optimizing oxygenation while minimizing VILI. Common approaches include: (1) selecting the PEEP that yields the highest static C_RS_ (thereby minimizing driving pressure); (2) targeting a positive P_tp_ee_direct_ to account for variability in lung and chest-wall mechanics, promote alveolar recruitment, and limit end-inspiratory P_tp_ to avoid excessive lung stress; and (3) Titrating PEEP by visualizing lung aeration and collapse by EIT.

Krebs et al. showed that in 13 patients with moderate-to-severe ARDS, PEEP set either at maximal static C_RS_ or according to the ARDS Network table failed to prevent negative P_tp_ee_direct_; in fact, under compliance-guided PEEP, 50% of patients had P_tp_ee_direct_< 0 cm H₂O (20). Suarez-Sipmann et al. (21) monitored dynamic C_RS_ and the percentage of non-aerated tissue on chest CT during PEEP titration following RM in an animal model with repeated lung lavages. They found that the proportion of non-aerated tissue increased during the decremental PEEP trial, even at the PEEP level associated with the highest dynamic C_RS_. According to each protocol, further analysis of individual PEEP data shows that each patient had different PEEP levels (19). These findings align with our results, which showed that OP differed significantly across the five targets. P_tp_ee_direct_ guided PEEP (≥ 0 cm H₂O) was higher than PEEP determined by the highest C_RS_ or C_L_.

Various protocols have been proposed to set OP in ARDS patients and mitigate VILI, specifically, overdistension and lung collapse (2). We characterized the potential risk of VILI—both overdistension and collapse—across different targeted PEEP levels. Overdistension was evaluated using surrogate markers: plateau pressure > 30 cm H₂O, P_tp_ei_derived_ > 25 cm H₂O, and the EIT-derived percentage of overdistension. Surrogates for collapse (atelectrauma) included negative end-expiratory P_tp_ values and the EIT-derived percentage of lung collapse.

P_tp_ee_direct_ guided PEEP (≥ 0 cm H₂O) met overdistension criteria—plateau pressure > 30 cm H₂O, P_tp_ei_derived_ approaching 25 cm H₂O, and the highest EIT-derived overdistension percentage. In contrast, C_RS_-guided PEEP carried a greater potential risk of collapse, evidenced by negative P_tp_ee_direct_ values and a higher EIT-measured collapse percentage. Additionally, P_tp_ee_direct_ was significantly inversely correlated with the EIT-derived collapse percentage. The underlying reasons for these findings are: (1) this approach yields the highest PEEP among the five methods; and (2) it is selected to maximize recruitment of dependent lung regions, reflected by the lowest EIT-derived collapse percentage. Figure 5 illustrates the relationship between P_tp_ee_direct_ and collapse percentage during PEEP titration; the P_tp_ee_direct_ associated with zero collapse on EIT was 5.15 cm H₂O (1.90, 6.70), a value consistent with Yoshida et al., who reported a minimum P_tp_ of 4.6 cm H₂O to prevent collapse by EIT assessment (18).

Costa et al. described an EIT-based method for assessing cumulative alveolar collapse and overdistension by analyzing regional pixel compliance during PEEP titration. They reported excellent agreement between recruitable collapse estimated by EIT and the increase in collapse relative to the minimum CT-determined collapse across all PEEP levels. Thus, EIT-guided PEEP appears to balance overdistension and collapse (10). In our study, the percentage of overdistension by EIT correlated significantly with peak airway pressure in PCV mode and with elastance-derived lung stress. However, because driving pressure remained constant in PCV, no correlation was observed between driving pressure and overdistension percentage.

Pavlovsky et al. compared PEEP titration strategies based on EIT, namely, center of ventilation closest to 50% and PEEP from balancing the degree of overdistention and lung collapse, and methods derived from respiratory system mechanics and P_tp_ monitoring. They showed that the different PEEP titration strategies led to differences in lung mechanics. The OP levels assessed by the crossing point method were higher than P_tp_ee_direct,_ which is contrary to our study. These differences may be explained by the method used to compute lung collapse and overdistension, particularly the comparison between maximal and current compliance at each pixel for a given PEEP level, and may be attributed to the application of PEEP levels during the decremental PEEP trial (from 20 to 0 cm H_2_O compared with from 20 cm H_2_O to 8 cm H_2_O in our study) (22).

## 7 Clinical implications

In practice, some methods yield higher PEEP levels—risking overdistension—whereas others produce lower PEEP levels with a potential for collapse. The relative harms of atelectrauma versus overdistension remain debated; however, experimental ARDS models suggest that volutrauma from excessive ventilation elicits a more pronounced inflammatory response than atelectrauma (23). On the other hand, in an RCT using a porcine model, EIT-guided strategies that minimized collapse —or explicitly balanced overdistension and collapse—were associated with lower mortality compared with approaches focused solely on preventing overdistension (24).

Grasso et al. used P_tp_ei_derived_ to adjust PEEP in severe H1N1-associated ARDS, increasing it until end-inspiratory Ptp reached 25 cm H₂O. This strategy significantly improved oxygenation and, in some patients, obviated the need for extracorporeal membrane oxygenation (25). In our study, P_tp_ei_derived_ guided titration yielded an optimal PEEP lower than that targeted by P_tp_ee_direct_, although the difference was not statistically significant. Repeated-measures ANOVA showed that P_tp_ei_direct_ values were consistently lower than P_tp_ei_derived_ (Supplementary Figure 2). Prior studies in porcine models and human cadavers have demonstrated that P_tp_ei_derived_ closely approximates inspiratory pleural pressure in non-dependent lung regions (18). The P_tp_ei_derived_ represents the highest level of inspiratory lung stress and can be considered a clinical target for minimizing VILI. This approach is consistent with the stress–strain concept, suggesting that P_tp_ei_derived_ guided PEEP selection or tidal volume reduction may lower the risk of volutrauma. Meanwhile, maintaining a P_tp_ee_direct_ ≥ 0 cm H_2_O helps prevent atelectrauma by reducing lung collapse (26).

An ideal PEEP strategy should: (1) ensure adequate gas exchange; (2) maintain lung patency by keeping P_tp_ee_direct_ ≥ 0 cm H₂O; (3) prevent overdistension by limiting P_tp_ei_derived_ to ≤ 25 cm H₂O or by selecting the EIT-derived balance point between overdistension and collapse; and (4) preserve hemodynamic stability (27).

During PEEP titration, all EIT-derived overdistension percentages were analyzed. The median overdistension (147 measurements from 21 patients) was 12% (2, 23). Patients were categorized into two groups: those with EIT-derived overdistension ≥ 12% were defined as having a higher percentage of overdistension, and those with < 12% were expressed as a lower percentage. We further analyzed the best threshold value using discriminant analysis. The threshold value of peak airway pressure that best distinguishes these two groups of patients was 30 cm H_2_O, and the best threshold value of elastance-derived lung stress was 25 cm H_2_O. Likewise, we subgrouped patients according to the threshold value of peak airway pressure by 30 cm H_2_O and elastance-derived lung stress by 25 cm H_2_O. We found a similar best threshold value of the overdistension at 12 percent. As a result, we may use 12 percent of overdistension by EIT to identify the higher percentage or the lower percentage of overdistension.

## 8 Limitations

This study has limitations. The sample size was small and predominantly comprised patients with intrapulmonary ARDS. In ARDS with normal chest-wall mechanics, Ecw contributes roughly 15–20% of Ers; our median Ecw/Ers was 0.18 (0.11, 0.27), so the findings may not extrapolate to patients with elevated chest-wall elastance. All measurements were obtained in the supine position; prone positioning might yield different results.

In our study, PEEP was reduced from 20 cm H₂O to 8 cm H₂O in 2 cm H₂O decrements at one-minute intervals, which may not allow full stabilization of respiratory system mechanics and may explain the absence of hemodynamic instability. During PEEP titration, the interval at each PEEP level is commonly about 2 min, but protocols vary: some extend to 3 or even 10 min, whereas others shorten the interval to approximately 30 s (19, 28, 29). A decremental PEEP trial generally requires a shorter equilibration time than an incremental trial. In a cohort of 44 ARDS patients, when PEEP was reduced (e.g., from 15 to 10 or from 15 to 5 cm H₂O), oxygenation variables stabilized within 5 min, while respiratory system compliance declined only slowly and modestly over 60 min (29). However, experimental and clinical observations indicate that a dwell time as short as ~40 s can still yield a reasonably accurate estimate of changes in compliance, because any airway closure during a decremental PEEP trial occurs very rapidly (30, 31). Finally, this study was not designed to assess clinical outcomes, and final PEEP settings were determined by the attending physicians. Further research is warranted to evaluate whether optimized PEEP-titration strategies that reduce VILI risk translate into improved clinical outcomes.

## 9 Conclusion

In 21 patients with moderate–severe ARDS, OP varied significantly across five titration methods, with median values as follows: 8.0 cm H₂O (8.0, 13.9) by C_RS_, 9.8 cm H₂O (8.0, 14.0) by C_L_, 14.0 cm H₂O (11.9, 17.9) when targeting P_tp_ee_direct_ ≥ 0 cm H₂O, 12.0 cm H₂O (10.0, 13.9) when limiting P_tp_ei_derived_ ≤ 25 cm H₂O, and 13.01 cm H₂O (9.88, 14.78) by the EIT crossing-point method. P_tp_ee_direct_ guided PEEP produced the greatest overdistension (median plateau pressure 31.3 cm H₂O [27.7, 33.3]; EIT overdistension 17% [6, 22]), whereas C_RS_-guided PEEP carried the highest collapse risk [median P_tp_ee_direct_ −2.3 cm H₂O (−4.2–0.8); EIT collapse 13% (6, 21)]. These findings underscore that OP is method-dependent and that effective titration must balance recruitment against the risks of overdistension and collapse.

## Data Availability

The raw data supporting the conclusions of this article will be made available by the authors, without undue reservation.
