# Supplementary material for: The potential risk of ventilator-induced lung injury from five different PEEP titration techniques in ARDS
Source: Front Med (Lausanne). 2025 Aug 29;12:1642064. doi: 10.3389/fmed.2025.1642064 (PMC12425713; doi:10.3389/fmed.2025.1642064)
Supplement: Supplementary file 1 [file Data_Sheet_1.docx]

Supplementary Material

**Supplementary Figure 1**. The Electrical impedance tomography (EIT)EIT analysis shows the percentages of alveolar collapse and overdistension.


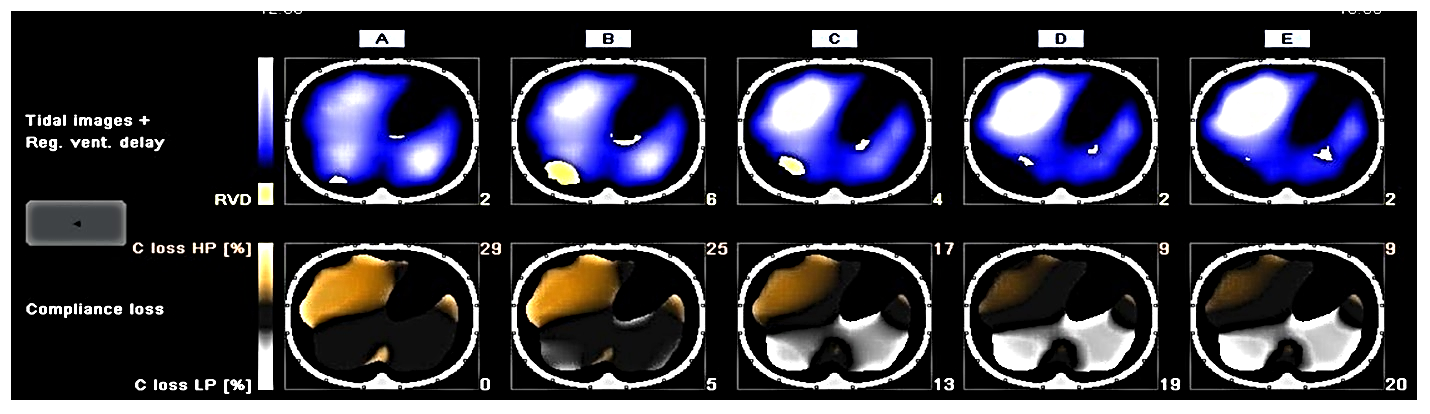


The picture for the EIT analysis shows the evolution of overdistension (orange zone) and the collapse region(white zone) during decremental PEEP titration from 20 to 12 cmH2O

[A: positive end-expiratory pressure (PEEP) 20 cmH2O; B: PEEP 18 cmH2O; C: PEEP 16 cmH2O; D: PEEP 14 cmH2O; E: PEEP 12 cmH2O]. The optimum PEEP level from EIT analysis would be PEEP level C, which balances the degree of overdistension and lung collapse.

**Supplementary figure 2**. The figure shows the statistical difference between end‑inspiratory transpulmonary pressure from direct measurement(P_tp_ei_direct_) and end‑inspiratory transpulmonary pressure from the elastance-derived method(P_tp_ei_derived_), p<0.001 by repeated-measure ANOVA.


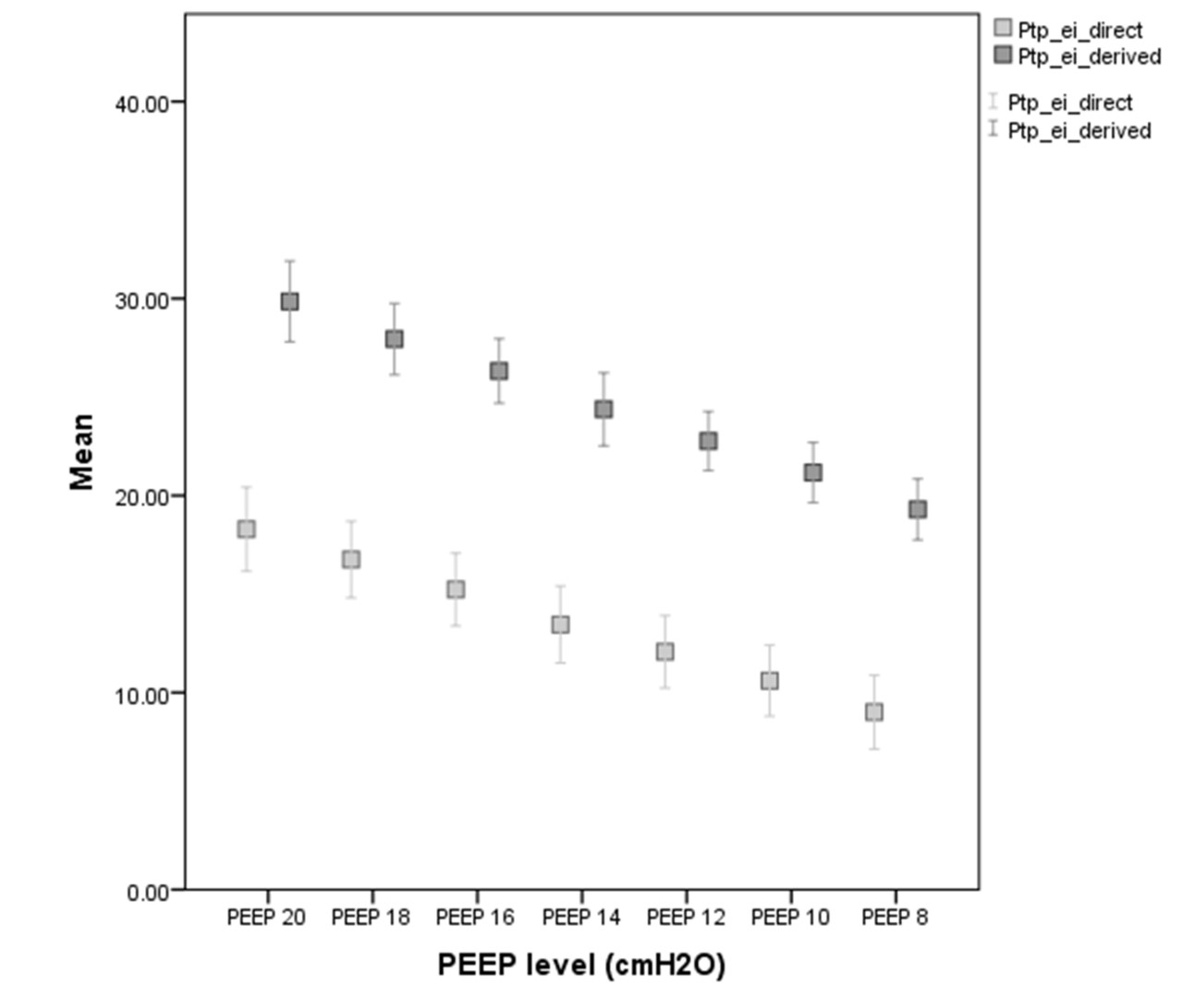


**Supplementary Table 1**: Hemodynamic parameters and SpO2/FiO2 ratio during decremental PEEP titration

| **Parameters** | **PEEP 20 cmH_2_O** | **PEEP 18 cmH_2_O** | **PEEP 16 cmH_2_O** | **PEEP 14 cmH_2_O** | **PEEP 12 cmH_2_O** | **PEEP 10 cmH_2_O** | **PEEP 8 cmH_2_O** |
| --- | --- | --- | --- | --- | --- | --- | --- |
| SBP, mean±SD, mm Hg | 124.8±15.3 | 126.1±15.8 | 126.9±15.8 | 127.5±15.0 | 126.5±15.0 | 126.3±14.9 | 126.3±14.1 |
| DBP, mean±SD, mm Hg | 66.0±7.7 | 65.0±7.3 | 64.8±7.3 | 63.9±7.5 | 64.1±7.6 | 63.5±7.7 | 63.3±7.5 |
| MAP, mean±SD, mm Hg | 85.61±8.25 | 85.36±7.99 | 85.52±7.58 | 85.09±6.42 | 84.88±6.36 | 84.39±7.33 | 84.27±6.95 |
| HR, mean±SD, beat/minute | 89.4±20.6 | 87.5±21.0 | 88.7±21.6 | 88.7±22.5 | 88.4±23.3 | 89.6±23.5 | 88.1±23.9 |
| CO, mean±SD, L/minute | 4.7±1.3 | 4.7±1.3 | 4.8±1.4 | 5.0±1.8 | 5.0±1.8 | 5.1±1.9 | 5.3±2.0 |
| SV, mean±SD, ml. | 53.85±19.74 | 55.66±18.97 | 55.41±19.53 | 57.47±22.09 | 58.12±22.65 | 58.33±22.65 | 61.66±23.30 |
| PPV, mean±SD, % | 13.7±7.4 | 10.6±5.4 | 10.3±5.8 | 9.5±6.3 | 9.0±5.9 | 8.6±5.0 | 8.4±3.8 |
| SpO2/FiO2 ratio mean±SD | 153.36±57.89 | 153.87±58.44 | 154.49±59.29 | 154.67±59.22 | 154.97±59.11 | 154.37±59.36 | 152.99±59.79 |

Abbreviations: SpO2, peripheral oxygen saturation; FiO2, fraction of inspired oxygen; SBP, systolic blood pressure; DBP, diastolic blood pressure; MAP, mean arterial pressure; HR, heart rate; PPV, pulse pressure variation; SV, stroke volume; CO, cardiac output
